# Current Insights on Bioactive Molecules, Antioxidant, Anti-Inflammatory, and Other Pharmacological Activities of Cinnamomum camphora Linn

**DOI:** 10.1155/2022/9354555

**Published:** 2022-10-07

**Authors:** Mohamed Joonus Aynul Fazmiya, Arshiya Sultana, Khaleequr Rahman, Md Belal Bin Heyat, Sumbul, Faijan Akhtar, Salabat Khan, Seth Christopher Yaw Appiah

**Affiliations:** ^1^Department of Amraze Niswan wa Ilmul Qabalat, National Institute of Unani Medicine, Ministry of Ayush, Bengaluru, Karnataka, India; ^2^Department of Ilmul Saidla, National Institute of Unani Medicine, Ministry of Ayush, Bengaluru, Karnataka, India; ^3^IoT Research Center, College of Computer Science and Software Engineering, Shenzhen University, Shenzhen, Guangdong 518060, China; ^4^School of Computer Science and Engineering, University of Electronic Science and Engineering, Chengdu, China; ^5^Health and Social Care Research Group, Department of Sociology and Social Work, Kwame Nkrumah University of Science and Technology, Kumasi, Ghana

## Abstract

*C. camphora* is a renowned traditional Unani medicinal herb and belongs to the family Lauraceae. It has therapeutic applications in various ailments and prophylactic properties to prevent flu-like epidemic symptoms and COVID-19. This comprehensive appraisal is to familiarize the reader with the traditional, broad applications of camphor both in Unani and modern medicine and its effects on bioactive molecules. Electronic databases such as Web of Science, PubMed, Google Scholar, Scopus, and Research Gate were searched for bioactive molecules, and preclinical/clinical research and including 59 research and review papers up to 2022 were retrieved. Additionally, 21 classical Unani and English herbal pharmacopeia books with ethnomedicinal properties and therapeutic applications were explored. Oxidative stress significantly impacts aging, obesity, diabetes mellitus, depression, and neurodegenerative diseases. The polyphenolic bioactive compounds such as linalool, borneol, and nerolidol of *C. camphora* have antioxidant activity and have the potential to remove free radicals. Its other major bioactive molecules are camphor, cineole, limelol, safrole, limonene, alpha-pinene, and cineole with anti-inflammatory, antibacterial, anxiolytic, analgesic, immunomodulatory, antihyperlipidemic, and many other pharmacological properties have been established in vitro or in vivo preclinical research. Natural bioactive molecules and their mechanisms of action and applications in diseases have been highlighted, with future prospects, gaps, and priorities that need to be addressed.

## 1. Introduction

The WHO stated that around 80% global population uses different types of traditional medicine to treat many diseases [1]. Traditional medicine is the knowledge, skills, and procedures that indigenous peoples and other cultures have used for a long time in order to preserve health and avoid illness [2, 3]. One of the oldest forms of medicine is Unani medicine, which originated in Greek [4] and is based on seven essential factors responsible for the maintenance of health and imbalance any one of them can lead to disease or even to death. The great physicians, Avicenna and Galan, stated that the primary elements contribute to the formation of things in nature [5]. The intermixture of primary elements forms temperament. The temperament indicates the state of equilibrium to the number of elements and the ratio of the particular compound and different combination responses to its specific nature. Hence, any changes in the quality or quantity of humor alter the equilibrium and disturb the normal temperament. Proper physiological functions are maintained by the homeostasis of temperament. Simple temperamental imbalances cause intrinsic power (immunity) to fight back and maintain body normal levels. If the body's temperament, functions are impaired owing to a change in humor, diet therapy, pharmacotherapy, regimental therapy, or surgery may be required, depending on the situation [5, 6]. Temperament (*Mizaj*) and humor, which is the principal concept in this system and disturbances in the quality and quantity of humor, cause numerous conditions. The four main modes of therapy available in Unani medicine [7] are regimental diet therapy, pharmacotherapy, and surgery. Correction through pharmacotherapy and regimental therapy helps to maintain homeostasis of the humor [4]. First-degree drugs are safer, temperamental quality is less, and second-degree drugs are also safe and have strong temperamental quality but no toxic effect. Third-degree temperamental medicine drugs are strong, the toxic effect may manifest prominently, and fourth-degree drugs are excessively strong and toxic. The drug is moderate (*Mutadil*) in temperament; it has no toxic, no temperamental quality, and the activity is only limited medicinal effect. Commonly, cold temperamental drugs are suitable for hot temperamental individuals and may produce effects on cold temperamental individuals in inappropriate doses. *Cinnamomum camphora* (L.) is a traditional Unani medicinal plant with a third-degree cold and dry temperament and hence useful in hot temperamental individuals [8].

*C. camphora* (L.) is a renowned Unani medicinal herb applied for several disease conditions in Unani as well as other traditional medicines. Camphor is a terpene ketone derived from *C camphora* wood or synthetically produced from turpentine. White, yellow, brown, and blue camphor oil are the four different fractions of camphor oil [9–11]. Camphor is obtained by distillation with water from the wood of trees or plants and purified by sublimation, and it occurs in translucent white crystals [12–14]. Since the ancient era in the Unani traditional system of medicine, *C. camphora* has been using its ethnomedicinal properties like antiseptic, analgesic, and rubefacient properties. Camphor has been use for very long time in various traditional systems of medicine such as Ayurveda, Unani, Siddha, and Chinese. It has been used in Unani medicine mainly in respiratory disorders *(Amrāz-i-Riyah)*, gastrointestinal (*Amrāz-i-Me‘da wa Am'a*), integument disease *(Amrāz-i-Jild*), *eye diseases (Amrāz-i-Ayn)*, and nervine and cerebral disorders *(Amrāz-i- damaghi wa a'sabi*) especially in hot conditions for headache, strengthening senses and brain [15], bilious diarrhea [8], inflammation of the liver [15], and useful in bladder and kidney inflammation [16]. Furthermore, externally, it is used for various ailments such as eye diseases, ear pain, joint, muscular pain, chest congestion, and headache applications such as ear drops or gargling with or without other suitable drugs [12, 15, 16]. An overdose may result in systemic toxicity. Signs of intoxication include gastrointestinal pain, emesis, agitation, tremors, and convulsions, which are followed by CNS depression marked by apnea and coma [17]. Furthermore, it is an important ingredient of *Arq Ajeeb* used as a prophylactic medicine for COVID-19 as per AYUSH guidelines. This comprehensive appraisal is to familiarize the reader towards the extensive, well recognized, and broad applications of camphor both in Unani and contemporary applications.

**Figure 1:**
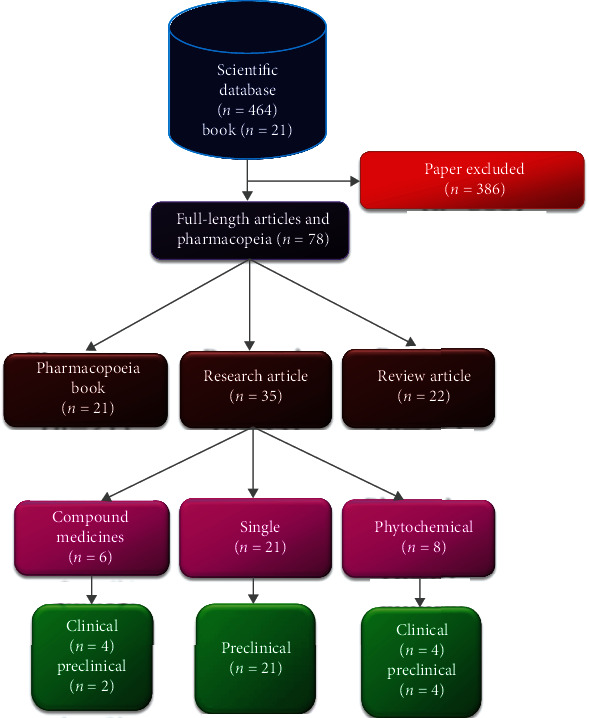
Flow diagram of type and inclusion and exclusion of the study.

To collect information on *C. camphora* for its temperament (*Mizaj*), adverse effects (*Mudir*), corrective (*Muslih*), substitute (*Badal*), ethnomedicinal properties (*Afa'l*), Unani compound formulations, and ethnomedicinal therapeutic uses, a literature survey of traditional Unani texts was conducted. Additionally, full-text paper and thorough search of electronic databases such as PubMed, Scopus, Google Scholar, and Research Gate were conducted to gather all accessible information on phytochemical, physicochemical, and pharmacological investigations relevant to *C. camphora*. All relevant articles are written in English up to 2022. The search occurred between August 2020 and May 2021. The keywords used were as follows: “*C. camphora*,” “chemical component,” “Unani Medicine,” “*Kafoor*,” “preclinical studies,” “clinical trial,” “phytochemical,” “adverse effect,” “toxicity,” and “traditional.” Chemical structure images were taken by PubChem. Standard Unani Medical Terminology of WHO was reviewed to define the suitable Unani terminologies. The scientific name and synonyms were authenticated and reproduced using The Plant List (http://www.theplantlist.org). A total of 464 papers and 21 books were retrieved, 386 were excluded, and we included research and review papers from the electronic database (Figure 1). Twenty-one included Unani classical manuscripts, and herbal pharmacopeial texts were consulted, including the incorporation of Urdu translation of the traditional textbooks such as *Makhzan al-Mufridat*, *Al Jami ul Mufradat Al Advia Wal Aghzia* (1197-1248 AD), *Muhit-i-A'zam* (1806–1902 AD), *Khazainul Adwiya*, (19th century), and *Bustan ul-Mufridat*, *Kitab ul Mansuri* (850-925 AD).

We collected data from traditional, classical Unani and herbal pharmacopoeia literature and up-to-date reviews and research to address a traditional and contemporary overview of the application of *C. camphora* in various ailments. We conducted this review to report most of the information on *C. camphora* therapeutic and traditional uses for several diseases. In addition, we also included a comparison between therapeutic traditional uses and its current research to prove its ethnomedicinal properties. Furthermore, the mechanism of natural bioactive molecules isolated from *C. camphora* was also highlighted. Preclinical and clinical trials were also reviewed to prove the effect of *C. camphora* in various diseases. Because no previous publications have incorporated this type of information in the review article, this review aims to overview and analyze the taxonomy, distribution, macroscopic description of the plant, various ethnomedicinal properties, therapeutic Unani applications, natural bioactive molecules isolated from different parts of the plant, present mechanism of action of natural bioactive molecules, comparison of therapeutic Unani applications proven currently by preclinical and clinical studies, gap, and future recommendation.

Hence, the following are the primary contributions of this study:

- Traditional Unani overview of *C. camphora* plants such as temperament, ethnomedicinal properties, therapeutic applications in various disease conditions, adverse effects, corrective, substitute, and Unani compound formulations containing *C. camphora* with dose and therapeutic applications
- Various natural bioactive molecules separated from various parts of the *C. camphora* plant with their structures and their mechanisms of action depiction such as analgesic, anti-inflammatory, antioxidant, and anti-allergic
- We also highlighted current research carried out in vitro, in vivo, and silico pharmacological studies such as antioxidant, anti-inflammatory, anti-allergic, antibacterial, antifungal activity, anxiolytic and antidepressant, analgesic, anti-hyperlipidemic, antifertility, hepatoprotective, antifertility, wound healing, prostaglandin synthesis inhibition, and oestrogenic activities
- We included preclinical and clinical studies of the main active biomolecules to report the significant use of C. *camphora* in day-to-day life. It also has a prophylactic effect against the SAR-CoV virus as per the study, and it is one of the main ingredients of *Arq Ajeeb* compound formulation used as a prophylactic inhaler to prevent COVID-19 infection
- This review also contributes toward comparative therapeutic evaluation, research gaps, future recommendations, and conclusions

## 2. Vernacular Name, Taxonomy, Distribution, and Types of *C. camphora*

The vernacular name summarized in Table 1. Camphor tree is a shrub or an evergreen tree [18] belonging to the family Lauraceae, Laurales order, genus Cinnamomum, and species *camphora* [14, 19]. Over 250-300 species of the genus are distributed globally [20]. Twenty-six species are found in India, and approximately 40 species are commonly used for medical conditions. Leaves and stem bark are the sources of medicinal activity. *Cinnamomum zeylanicum*, *C. camphora*, *C. burmannii*, *C. cassia*, *C. tamala*, and *C. verum* species are rice sources of aromatic oil [20, 21]. Camphor (*Kafoor*) is an exudate of a camphor tree as per the description in Unani literature. Moisture or liquid comes out from cracks or incision in the tree and freezes out as rust. It is also expelled through the hole of the tree [12], or the wood of that tree is chopped and soaked and heated in the water causing sublimation [12]. It is clear crystal white with a strong smell [8, 12]. Camphor is found naturally or artificially synthesized. The natural camphor is D-camphor, whereas the synthetic one is L-camphor [22]. Authentic Unani texts described numerous varieties of camphor. The best type is *Kaisuri* followed by *Riyahi. Kaisuri* is found in the city of *Qaisr* on the island of *Tarindib*, so the name has been given *Kafuri Kaisur* [8]. There are three different kinds of camphor: Formosa camphor [22], Borneo or Barus camphor, and blumea or Ngai camphor. Borneo camphor is high priced, and it is naturally formed in the stems of Dryobalanops camphor grown in Dutch Sumatra and sinks in water. This is considered the best type. Borneo Camphor is high priced, and it is naturally formed in the stems of *Dryobalanops camphor* grown in Dutch Sumatra and sinks in water, and the third type is Blumea or Ngai camphor.

## 3. Macroscopic Description

Its opposite, frequently three-nerved, long petiolate, oblong or ovate, 5- to 12.5-cm long, and 2.5 to 5-cm broad leaves are usually three-nerved. Its flowers are tiny and hermaphrodite or produced via polygamy or abortion. Typically, females are larger and have few components. Nine stamens are there, unless they are aborted. The ovary is sessile, free from the perianth, style is narrow, the stigma is discoid, and the style is narrow or obscurely 3-lobed [18]. The fruit is a berry with spreading, somewhat expanded perianth, completely or partially deciduous segments, and less frequently persistent seeds [18].

## 4. Description in Unani Literature

### 4.1. Temperament (Mizaj)

Temperament is one of the unique features and fundamental principles of the Unani system of medicine [24]. All medicinal substance, plants, animals, and minerals have their temperament. The temperament of drugs is used as a tool to assess the actions and toxicological properties of Unani drugs. The medications were divided into four groups based on their innate nature: hot (*Hārr*), cold (*Bārid*), moist (*Ratb*), and dry (*Yābis*) in terms of their effect on a moderate human body and four degrees 1, 2, 3, or 4 in terms of increasing intensity of action. As a result, different scholars have claimed that its temperament is variable [12]. The temperament of camphor is cold and dry in the third degree [12, 14], cold second, and dry in the third-degree [8].

### 4.2. Ethnomedicinal Properties (Afa'l)

**Figure 2:**
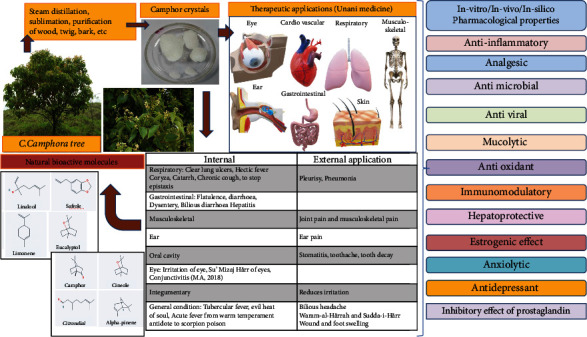
Overview of ethnomedicinal properties, therapeutic applications in Unani medicine, and pharmacological activities.

Camphor, administered orally, has several pharmacological properties such as expectorant (Munaffith-i-Balgham) [12]; stimulant (Muharrik) [12]; brain and heart tonic (Muqawwi-i-Qalb wa Dim$\overset{¯}{a}$gh) [12]; exhilarant of brain and heart (Mufarrih al- Qalb wa Dim$\overset{¯}{a}$gh) [15]; antipyretic (D$\overset{¯}{a}$fi-i-Humm$\overset{¯}{a}$) [12, 16]; hemostatic ($\underset{˙}{H}$$\overset{¯}{a}$bis-i-Dam); anti-pyretic for tubercular infection ($\underset{˙}{H}$umm$\overset{¯}{a}$ Diqq) (a form of fever that gradually depletes the body's fluids and weakens its organs, resulting in weight loss) [12, 16]; antispasmodic (D$\overset{¯}{a}$fi?-i-Tashannuj) [12]; astringent (Q$\overset{¯}{a}$bi$\underset{˙}{d}$) [12]; disinfectant (Mani'-i-'Ufunat) [15]; anaphrodisiac (Duf-i-Bah- [12]; constipation (Qabz) [12]; and carminative, reflux expectorant, which stimulate the heart and respiratory system and analgesic and sedative to the nervous system [13]. Additionally, the overview of ethnomedicinal properties, therapeutic applications in Unani medicine, and pharmacological activities are mentioned in Figure 2 (https://www.ayurtimes.com/cinnamomum-camphora/3/6/2022 and https://plant.ces.ncsu.edu/plants/cinnamomum-caomphora/).

#### 4.2.1. Topical Application

Topical application of camphor has antiseptic [12] and massages externally and initially; it has stimulant [12] rubefacient (Muhammir) and then anesthetic (Mukhaddir) [12] and analgesic (Musakkin-i-Alam) ethnomedicinal properties [12, 16].

### 4.3. Therapeutic Application as per Unani System of Medicine

As per Unani system of medicine, Camphor is useful in respiratory, gastrointestinal, musculoskeletal, integumentary, oral cavity, ear condition, eye diseases, and other general conditions (Table 2).

## 5. Therapeutic Dose, Adverse Effects, and Correctives of *C. camphora* in the Traditional and Contemporary Era

Unani classical texts mention doses range from minimum to 182 [12] up to 250 mg to 550 mg. According to another opinion 7 gm/week [15], the maximum dose is 364 mg to 728 mg. More than 8.75 gm reduces sexual power or may cause death [16]. The minimum dose is 182 to 364 mg and can be given for strengthening the patient [16]. The lethal dose in adult humans is 5 to 20 g. One teaspoon of camphorated oil (~1 mL of camphor) was lethal to 16 and 19-month-old children [25]. Unani scholars stated that overdose or misuse of this drug may adversely affect cold temperamental people (*Barid Mizaj)* [12, 15, 16], stagnation of sperm (*Munjamid Mani*), and weakness in individual's stomach [15, 16] that reduces sexual power and sperm quality and forms kidney stone [12, 15, 16].

Camphor is quickly absorbed by the mucous membranes, skin, and gastrointestinal tract in liquid form. Symptoms may appear 5–90 minutes after consumption. The rate of absorption is heavily reliant on the existence of food and other compounds [26]. In humans, intoxication signs are abdominal distress, emesis, tremors, excitement, seizures, and CNS depression characterized by apnea and *coma* [17, 25]. The traditional Unani medicine discusses various corrective agents (*Muslih*) and forms of simple and compound drugs that can use according to the condition internally or externally to manage or minimize the adverse effects of camphor. As camphor is cold temperament and to combat it coldness, hot and aromatic herbs such as oil of *Viola odorata* L. (*Rogan-i-Banafsha*), *oil of Narcissus tazetta* Linn (*Rogan-i-Nargis*), *Ambar*, and Castoreum (*Jundabedastar*) [15, 16] are used as corrective. Oil of *Iris ensata* Thunb (*Roghan-i-Sosan*), [12, 16] a flower of *Viola odorata* L. (*Gul-i-Banafsha*) [12], and a confection of *Rosa damascene* act as a corrective in a condition such formation of renal stone caused by camphor use [15]. *Narcissus tazetta* L. (*Banafsha*), *Nelumbo nucifera* Gaertn (*Niloufer*), *Crocus sativus*, a confection of *Rosa damescene* (*Gulkand*), *Ambar*, and *Musk* are drugs that act as corrective in headache caused by use of camphor [15].

## 6. Substitute (*Badal*)

The idea of drug substitution (*Abdāl-i-Adwiya*) is a significant criterion of Unani pharmacotherapy [27]. In Unani Medicine, replacing the main drug with a substitute having the same or closest pharmacological action with the first desired drug and a substitute can be chosen depending on the situation. Need for drug substitutes, Al-Razi surmised that “frequently, all drugs needed for treatment are not easily available everywhere. As a result, if a physician is uninformed of the replacements that must be used in place of the principal drug, the medical profession's objectivity will be compromised” [28]. The great physician, Avicenna states that a substitute can be used “When the initially intended medicine is unavailable” [4, 29]. In case of non-availability of Camphor, *Barbarea vulgaris* (*Tabashir Sufaida*) [12, 16, 30] or *Pterocarpus santalinus* (*Sandal*) [15, 16] or fossil resin of *Pinus succinifera* (*Kahruba*) [15] can use as a substitute.

## 7. Ethnomedicinal Properties and Therapeutic Applications of Unani Compound Formulations of Camphor (*Murakkabat*)

Compound medicines are pharmaceuticals that contain two or more herbs as ingredients in a variety of dose formulations and administration routes. Topical preparations include ointment, lotions, and fine powders for ocular use. Oral preparations include pills, tablets, powder, and semisolid confection forms. Several Unani compound formulations as per pharmacopoeia preparation possessing different ethnomedicinal properties, therapeutic applications, and dosage forms with doses acting on different body systems have been described in detail in Table 3.

## 8. Natural Bioactive Molecules of *C. camphora* and their Mechanism of Action

**Figure 3:**
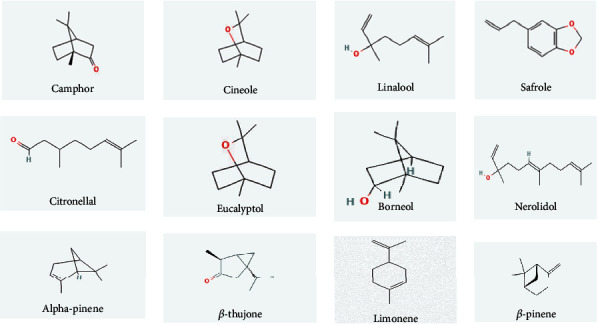
Bioactive molecules in *C. camphora.*

Seventy-four compounds were discovered in leaf, branch, wood, and root chromatograms of *C. camphora* tissues [35]. Phytochemical components of *C. camphora* are phenolics, flavonoids such as tannins (2.09%), saponins, alkaloids (3.85%), and carbohydrates [36]. The bioactive compound of *C. camphora* oil identifies the relevant analgesic effects *β*-caryophyllene, *α*-caryophyllene, germacrene D, bicyclogermacrene, unidentified, nerolidol, spathulenol, and unidentified (E)-*α*-atlantone [36]. Another study identified 96 various compounds in the essential oils by two-dimensional gas chromatography such as methyl isobutyl ketone, pinene (*α* and *β*), *α*-thujene, camphene, sabinene, *α*-phellandrene, hexanal, 3-hexanal-1-ol, 1-hexaol, and sabinene [37]. The major bioactive molecules of *C. camphora* are camphor, linalool, safrole, and cineole. The major bioactive molecules in different parts of the plant are mentioned in Table 4 and Figure 3.

The pathophysiology of respiratory diseases (sinusitis, asthma, bronchitis, and COPD) is mucociliary dysfunction, inflammation-induced edema, and hypersecretion of goblet cells that probably plays an important role. Cineole natural substance from camphor has pharmacological properties that are known to reduce inflammation, secretion of goblet cells decreases, the ciliary beat frequency is sped up, and bronchodilatory and mucolytic properties hence help in the drainage of sinuses and other respiratory organs [3]. Consequently, cineole would be therapeutically beneficial for asthma and bronchitis patients based on its proven broncho-dilating and anti-inflammatory effects. Other studies also have proven the effect on cineole [41–44].

A new elucidation for the analgesic application of camphor is the combination of transient receptor potential A1 (TRPA1) inhibition and desensitization of TRPV1. Camphor activates and then desensitizes TRPV1, thereby having an analgesic action. Linalool bioactive molecules showed pain reduction in mouse models such as inflammatory pain, acetic acid-induced writhing response, and the hot plate test. The likely mechanism perhaps is related to its regulation of NMDA receptors and suppression of pro-inflammatory cytokines [36]. In a clinical investigation, topical borneol treatment dramatically reduced pain compared to placebo. Furthermore, an in vivo study in mice that exhibited TRPM8 channels may perhaps be the molecular target of borneol [45]. *C. camphora* natural bioactive molecule menthol after topical application (skin, mucous membrane, and oral and nasal cavities) also activates TRPA1, s highly sensitive menthol receptor that contributes in counterirritants and analgesic activities. This suggests the involvement of different kinetics of channels and fast desensitization due to these sensory effects of menthol [46].

**Figure 4:**
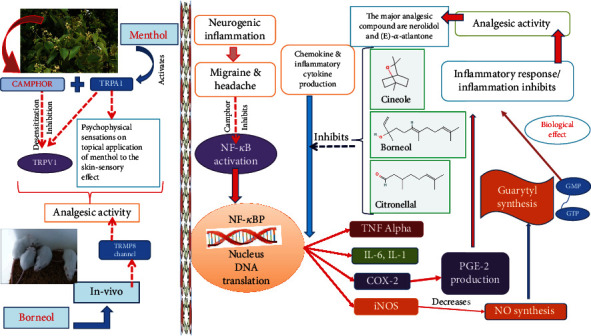
Anti-inflammatory and analgesic mechanisms of action of natural bioactive molecules of *C. camphora.*

Clinical investigations and contemporary medical experiments in migraine and vascular headache demonstrated the crucial role of nitric oxide (NO). NO and nitric oxide synthase (NOS) inhibitors can significantly reduce the severity, frequency, intensity, and accompanying symptoms of migraine attacks. A protein complex known as nuclear factor-kappa β(NF-B) regulates DNA transcription, cytokine synthesis, and cell viability. NF-*κ*B induces the expression of inducible nitric oxide synthase (iNOS), and overexpression of iNOS can catalyze L-arginine to yield nitric oxide (NO). Proinflammatory factors are also activated by NF-*κ*B and then cause a neurogenic inflammation reaction, which sensitizes the pain center and initiates headache. In the pathogenesis of migraine, NF-*κ*B has a significant mediating role. The essential oil of camphor leaves in the mouse model showed noteworthy analgesic action on migraine by inhibiting the nuclear factor-kappa B (NF-*κ*B)/inducible nitric oxide synthase (iNOS) pathway and reduced neurogenic inflammation. Essential oil of *C. camphor* perhaps inhibits the iNOS and expression of NF-*κ*B and therefore decreases NO production and neurogenic inflammatory response (Figure 4). Therefore, it could treat migraine. The main analgesic compounds recognized in the camphor's essential oil camphor leaves were nerolidol and (E)-*α*-atlantone [36]. Citronellol, which affects cyclooxygenase (COX) 1 and 2, is the enzymes involved in the production of prostaglandins from arachidonic acid and decreases the production of inflammatory mediators which is related to its ability to reduce cell migration and paw edema [47].

Markel et al. [48] investigated the influence of camphor on the expression of oestrogenic genes. They discussed how the UV filter 4-methyl benzylidene camphor (4-MBC) is oestrogenic and interferes with the thyroid axis. They discovered that, in rats, exposure to 4-MBC altered the mRNA levels of ER-alpha, progesterone receptor (PR), preproenkephalin (PPE), and insulin-like growth factor-I (IGF-I) in the brain in a sex- and region-specific manner. Methanolic extract of *C. camphora* contains anti-inflammatory mechanisms that limit NO and PGE2 synthesis in LPS/IFN-activated macrophages and prevent the generation of TNF IL-6 and IL-1 from RAW264.7 cell [10]. The proanthocyanidins (PAs) in the leaves of *C. camphora* inhibit tyrosinase monophenolase and hence have been proven to have anti-tyrosinase activity. The PAs also showed strong antioxidant capacity with the ferric reducing antioxidant power (FRAP), scavenging 2,2-diphenyl-1-picrylhydrazyl (DPPH) and 1,2′-azino-bis (3-ethylbenzthiazoline-6-sulphonic acid) (ABTS) assays [49].

## 9. Preclinical and Clinical Studies of Main Active Biomolecules

The preclinical studies of the main natural active biomolecules are summarized in Table 5.  Table 6 summarizes the preclinical and clinical studies of the compound formulation of camphor.

### 9.1. In Vitro Pharmacological Properties

Numerous in vitro experimental studies show that antioxidant, antimicrobial, anti-inflammatory, and miscellaneous activities have been demonstrated by numerous research on *C. camphora*.

#### 9.1.1. Antioxidant Activity

**Figure 5:**
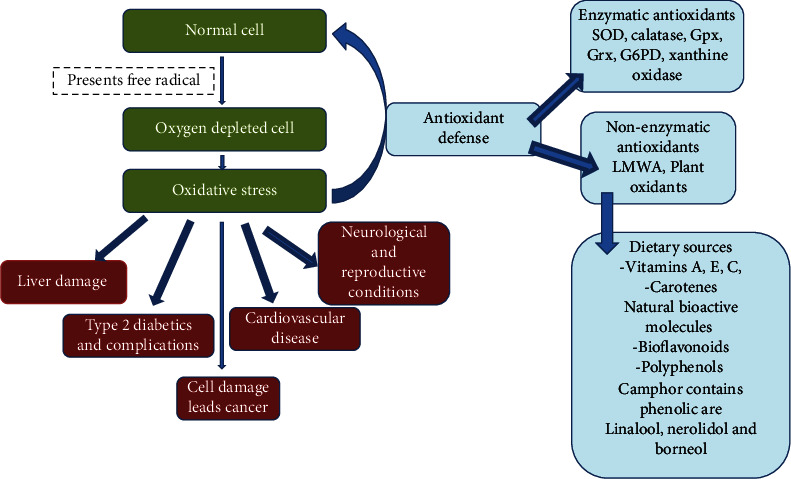
Oxidative stress affects various systems and antioxidant defenses including natural bioactive molecules.

**Figure 6:**
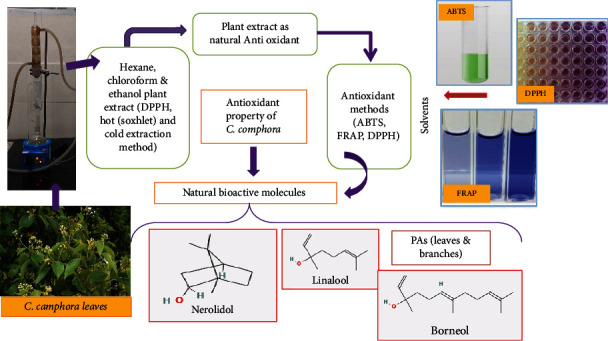
Antioxidant activity of natural bioactive molecules of *C. camphora.*

By interacting with biological components within the cell, the oxidation process damages cells, resulting in a variety of illnesses and chronic diseases like cancer and cardiovascular conditions. Additionally, oxidation changes the nutritional value and safety of food by producing secondary reaction products [58]. Oxidative stress is a condition when antioxidant levels are low. Antioxidant activity of polyphenols, which is influenced by their polyphenolic structure, has the effect of removing free radicals and improving antioxidant activity [59]. Due to an excess of reactive oxygen species (ROS), oxidative stress develops when the body's antioxidant system becomes depleted. Due to this, an increase in the concentration of free radicals inside cells is the root cause of many chronic disorders such as nonalcoholic fatty liver, type 2 diabetics, neurological conditions, and reproductive-related problems [59–61]. Reactive oxygen species (ROS) at baseline levels are necessary for basic physiological activities. Ageing, obesity, type 2 diabetes mellitus (T2DM), depression, and neurodegeneration are all conditions that are significantly impacted by oxidative stress [59] (Figure 5). Camphor has antioxidant, hepatoprotective, antidepressant, estrogenic, and anti-inflammatory qualities in addition to being an antioxidant that prevents oxidative damage and neutralizes free radicals. Camphor's antioxidant capabilities may lessen tissue damage and oxidative stress. In scavenging DPPH, ABTS, and ferric reducing antioxidant power (FRAP) assays, the phytochemical proanthocyanidins (PAs) from leaves and branches of *C. camphora* displayed significant antioxidant activity [49]. The antioxidant activity of hexane, chloroform, and ethanol extracts was determined using the DPPH (2,2-diphenyl-1-picrylhydrazyl) technique on dried camphor leaves. Another study evaluated the antioxidant activity of leaves of *C. camphora* in three different solvents and was tested by using the DPPH method, and hot extraction (Soxhlet) and cold extraction (maceration) methods were applied for the presence of components in the camphor leaves. The antioxidant activity of ethanol extracts was higher than that of other extracts. These findings show that camphor leaves, which have significant antioxidant quality, are excellent for pharmaceutical composition. Linalool, nerolidol, and borneol are the phenolic compounds extracted from the ethanolic extract. The hot extraction method by using ethanol solvent can extract antioxidant and mineral content against camphor leaves [62] (see Figure 6). Liu et al. [63] established the flavonoids extracted from C. camphora leaves' in vitro antioxidative capacity. Both the ferric reducing antioxidant power assay and the 1,1-diphenyl-2-picrylhydrazyl (DPPH) free radical scavenging assay demonstrated a dose-dependent increase in antioxidant activity in the flavonoids, which is outstanding compared to commercial antioxidants [64].

#### 9.1.2. Antimicrobial

Numerous disorders in the body are brought on by pathogens, which are inhibited by antimicrobial agents, which also stop the establishment of microbial colonies. One of the biggest problems in human health is the over and improper use of antibiotics. Additionally, the rapid spread of microorganisms that are resistant to antibiotics is concerning. The herbal potential of *C. camphora* is recognized to serve as an antibiotic, antiviral, and antifungal. Wang et al. [65] described that there is evidence that *C. camphora* essential oil has therapeutic properties like antibacterial effects. The obtained MICs and MBCs verified the clinical strains' significant susceptibility to CCEO. The development of E. coli biofilms is intimately associated to prolonged E. coli infection and can lead to antibiotic resistance. E. coli was significantly destroyed by CCEO, and the E. coli biofilm was also effectively destroyed. *C. camphora* essential oil (CCEO) was active against *E. coli* in suspension and biofilms, two states that are common in living organisms. Escherichia coli, one of the most frequent microbial pathogens, is mainly responsible for biofilm-associated opportunistic illnesses like diarrhea, endometritis, and mastitis [65]. In another in vitro study of camphor ethanolic extract has been showed antibacterial action against *Escherichia coli*, *Staphylococcus aureus*, and *Pseudomonas aeruginosa* [56]. Poudel et al. [37] analyzed in vitro study of *C. camphora* essential oil against five Gram-positive bacteria, *Streptococcuspyogenes*, *Propionibacteriumacnes*, *Bacilluscereus*, *Staphylococcusepidermidis*, and *Staphylococcusaureus*, and two Gram-negative bacteria, *Pseudomonas aeruginosa* and *Serratia marcescens*, showing its antibacterial properties [37].

The phytochemical compounds found in *C. Camphora* have a wide variety of antibacterial properties against various pathogens. Leaf, branch, and wood essential oil were tested again using seven strains of fungi, *Aspergillus niger*, *Aspergillus fumigatus*, *Candida albicans*, *Microsporum canis*, *Trichophyton mentagrophytes*, *Microsporum gypseum*, and *Trichophyton rubrum*. *Serratia marcescens* responded favorably to the wood essential oil's antimicrobial properties. Camphor, 1,8-cineole, -terpineol, and safrole were the main ingredients in the wood oil; hence, the reported activity of the wood oil against *S. marcescens* may be the result of synergism between these and other constituents. Only a small amount of action was seen against S. marcescens by camphor, 1,8-cineole, -terpineol, and safrole. According to one study, camphor and 1,8-cineole work together to have a synergistic antibacterial effect [35]. All fungi were cultured on yeast malt, and studies showed good antifungal activity against *Aspergillus niger* and *Aspergillus fumigatus*, while the leaf essential oil showed good antifungal activity comparatively to other parts of the plant [35]. Kulzam is a well-known Unani liquid composition used to cure several ailments such as cough, colds, and sore throats [66]. The major components identified in Kulzam were camphor, menthol, etc. The ingredients of the formulation are *Sat-i-Pudina*, *Sat-i-Ajwain*, *camphor*, *Roghan baid majnun*, *Roghan-i-darchini*, *Roghan-i-zaitun*, and *Roghan-i-laung*. Kulzam demonstrated a significant effect on all tested microorganisms at both 100 and 150 (micro) levels of the undiluted formulation (test sample), and at 150 (micro) level, it inhibited growth more than the standard. Furthermore, it highlights the fact that gram-negative microorganisms are more vulnerable to inhibitory activity than gram-positive ones. Comparing the formulation to that of standard clotrimazole, it showed a very significant zone of inhibition against the fungus *Candida albicans* and *Aspergillus fumigatus* [66]. In an in vitro investigation, essential oil from *C. camphora* leaves, flowers, and twigs showed antifungal action again 7 strains including *Aspergillus clavatus*, *Aspergillus niger*, *Chaetomium globosum*, *Cladosporium cladosporioides*, *Myrothecium verrucaria*, *Penicillium citrinum*, and *Trichoderma viride* in 1000 *μ*g/ml concentration [67]. In addition, when compared to other sections of the plant, the leaf oil exhibited the best antifungal efficacy [67]. Five locally gathered plant species' fresh leaves were hydro distilled using Clevenger's apparatus to separate the essential oils and stored in a glass jar. Using the poisoned food approach on a potato dextrose agar medium, the oils were evaluated for resistance to *Aspergillus flavus* at 5000 ppm. Only the oil of *C. camphora* showed absolute fungitoxicity against the test fungus among the five essential oils examined [55]. Studies showed camphor oil possesses mycostatic application against *Aspergillus flavu*s [68]. According to Karashima et al. [46], CHO cell showed induce expression of TRPA1, 0.5 mg/ml tetracycline was added to the culture medium, and cells were used 5–24 h after induction and menthol used as test drug*. C. camphora* constituent menthol activates TRPA1 and inhibits it in mouse neuron in in vitro study, suggesting the involvement of different kinetics of channel and fast desensitization due to these sensory effects of menthol, a widely used additive in counterirritants and analgesic activity.

#### 9.1.3. Anti-Inflammatory and Prostaglandin Synthesis Inhibition

Inflammation is a healing process that is triggered by pathogen, toxins, and radiations. These factors set off the immune system and cause inflammatory reactions in the organs of the host, which may result in cell death and/or illness. Unani traditional medicine is potentially useful for the treatment of inflammation-related diseases, such as rheumatism, bronchitis, asthma, COPD, acute non-purulent rhinosinusitis, dermatitis, neurodegenerative diseases, and muscle pains. There are well-known anti-inflammatory compounds that have been extracted from plants and evaluated in human clinical trials. Cinelol, cineole, citronellal, and camphor make up the majority of them [41–44]. Numerous investigations have revealed that *C. camphora* has an anti-inflammatory activity in vitro. An *in vitro* investigation of *C. camphora* leaf extract indicated that it reduced the generation of inflammatory chemokines. Its leaves had a significant impact on 2,4-dinitrochlorobenzene-induced atrophic dermatitis in mice. An *in vitro* investigation of *C. camphora* leaf ethanolic extract indicated that it reduced the generation of inflammatory chemokines. Its leaves had a significant impact on 2,4-dinitrochlorobenzene-induced atrophic dermatitis in mice. Lee et al. examined the inhibitory impact of CCex on IFN- (10 ng/mL) stimulated HaCaT keratinocytes' ability to produce the inflammatory chemokine (MDC). The outcomes demonstrated that CCex inhibited MDC formation by IFN- in a concentration-dependent manner. The MeOH extract of *C. Camphora* inhibited prostaglandin E2 (PGE2) production in LPS/IFN-activated macrophages by up to 70%. To further understand *C. camphora's* anti-inflammatory activity, researchers looked at macrophage-mediated inflammatory events like cytokine production, NO release, PGE2 release, functional activation of adhesion molecules, and oxidative stress. It can have a strong immunomodulatory influence on numerous inflammatory responses at the transcriptional level, according to the findings of the study [10]. Methanolic extract of *C. camphora* contains anti-inflammatory mechanisms that limit NO and PGE2 synthesis in LPS/IFN-activated macrophages and prevent the generation of IL-1, IL-6, and TNF- from RAW264.7 cells [10].

By interacting with biological components within the cell, the oxidation process damages cells, resulting in a variety of illnesses and chronic diseases like cancer and cardiovascular conditions. Additionally, oxidation changes the nutritional value and safety of food by producing secondary reaction products [64]. The effectiveness of EOC derived from leaves in treating allergic inflammation, such as atopic dermatitis, was described by Kang [69]. The extract significantly reduced inflammation in low-calcium, high-temperature human adult keratinocytes and improved 2,4-dinitrochlorobenzene-induced atopic dermatitis in mice. These results will make it easier to create EOC as a novel, all-natural treatment for inflammatory skin disorders [69].

#### 9.1.4. Anti-Hyperlipidemic Activity

Camphor compound was examined in rats with experimental dyslipoproteinemia for its pharmacotherapeutic efficacy, antioxidant, and anticoagulant action. The positive results of the study allowed this substance to be recommended for the prevention of atherosclerotic damage to the vascular endothelium and the prevention of thrombogenesis [70].

#### 9.1.5. Antifertility Activity

In order to understand the impact of camphor as a male local contraceptive, in-vitro effect of camphor on human sperm vialbility and motility was examined. A decrease in sperm motility and viability in an in vitro investigation where camphor was used indicates that fertilization efficacy is reduced. Camphor may work as a contraceptive effect. The sperm motility and viability decrease are probably because of a fall in fructose levels or denaturation of protein and cholesterol, which are the energy sources for sperm motility [71].

### 9.2. In Vivo Pharmacological Studies

#### 9.2.1. Wound Healing Activity

Camphor, a potent wound healing and ant wrinkle drug, reduced MMP1 expression but increased collagen and elastin expression in UV-exposed mouse skin after 4 weeks of therapy. Camphor might prevent the loss of elastin and help it recover after UV-induced damage to retain skin suppleness [60]. It also decreased the depths of the epidermis and subcutaneous fat layer in UV-exposed mouse skin. The ethyl acetate soluble fraction of an ethanolic extract of C. camphora leaves in Wister rats showed improvement in wound healing and increased wound contraction due to enhance and accelerated activity of fibroblast and epithelial cell migration to the wound site and early dermal and epidermal regeneration. Furthermore, the treated group also showed a considerable increase in collagen content [75].

#### 9.2.2. Anti-Testosterone Activity

Jugular vein samples were taken for hormonal analysis from Awassi lambs and rams fed *C. camphora* at a dose of 20 mg/kg/animal, and semen samples were collected from the animals using artificial vagina in the control group. The study found that the testosterone hormone concentration in the treatment group was much lower than in the control group, which could be attributed to camphor's oestrogenic impact, which reduces testosterone hormone levels. Camphor may suppress catecholamine secretion by inhibiting nicotine acetylcholine receptors, which has an influence on male sexual behavior and reproductively via its effect on blood testosterone levels and/or the sympathetic nervous system. During the second and fourth weeks of the experimental study, mass activity in the camphor group was significantly (*P* < 0.05) lower than in the control group, whereas the individual sperm motility percentage showed no significant differences between the camphor and control groups throughout the entire experimental period, i.e., over the final three weeks of the trial, the camphor group displayed lower levels testosterone [73].

#### 9.2.3. Oestrogenic Effect

Maerkel et al. [48] evaluated the estrogenic effect on the brain and reproductive organs both prenatal and postnatal exposure to UV filter 4-methylbenzylidene camphor (4-MBC) in rats. Following pre- and postnatal exposure to the UV filter 4-MBC, the current study found alterations in the expression level and estrogen sensitivity of target genes as well as in the steroid receptor coactivator SRC-1 in sexually dimorphic brain areas of adult rat offspring. They discussed how the UV filter 4-methyl benzylidene camphor (4-MBC) is oestrogenic and interferes with the thyroid axis. They found that 4-MBC exposure changed the mRNA levels of ER-alpha, progesterone receptor (PR), preproenkephalin (PPE), and insulin-like growth factor-I (IGF-I) at the brain level in rats in a sex- and region-specific manner [48].

#### 9.2.4. Anti-Allergic Activity

Edema, a dysfunctional skin barrier, and the invasion of several inflammatory cell types are the hallmarks of allergic skin inflammation, such as atopic dermatitis (AD). *In vivo*, *C. camphora* leaves (100 mg/kg) improved atopic dermatitis symptoms by lowering serum immunoglobulin E levels, reducing lymph node thickness and length, decreasing ear edema, and lowering the number of inflammatory cells infiltrating the ears. Atrophic dermatitis is an allergic inflammatory disorder that can be treated with the leaves of *C. camphora*. IFN-*γ*, an important mediator of immunity and inflammation, induces the Janus tyrosine kinases- signal transducer and activator of transcription (JAK-STAT) signal pathway. The investigators reported that in skin inflammation lesions, leaf extract inhibited macrophage-derived chemokine (MDC/CCL22) production via the downregulation of (STAT) 1 and extracellular signal-regulated kinase 1/2 (ERK1/2) pathways and, hence, improved several symptoms (ear edema and lymph node size) change in blood parameter (serum IgE) and histological changes in mice with allergic dermatitis. By administering DNCB to mice, we established experimental AD in order to research the effects of CCex on AD in vivo. IgE levels are correlated with the severity of AD and are linked to defective skin barrier, making IgE a key therapeutic target for AD. When compared to the induction group in this investigation, the CCex-treated group had considerably lower serum IgE levels (*p* 0.001). Comparing the cutaneous edema in the CCex-treated mice to that in the induction mice on day 29, the difference was significant (*p* 0.001). Additionally, the CCex-treated group showed considerably less epidermal thickness and inflammatory cell infiltration than the induction group [69].

#### 9.2.5. Anxiolytic and Antidepressant Activity

**Figure 8:**
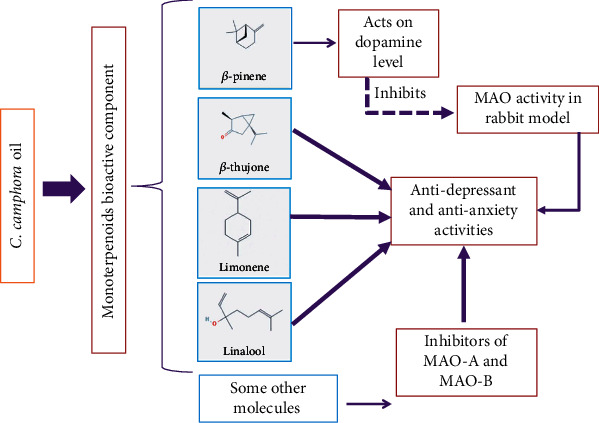
Anti-anxiety and antidepressant activity of natural bioactive molecules of *C. camphora*.

Antidepressant and anxiolytic medications are used to treat depression and anxiety. Albino mice weighing 18-30 gm were used for the study CCO given three different doses 250 mg/kg CCO orally, 500 mg/kg, and 750 mg/kg CCO orally in each group. and imipramine15mg/kg given intra peritoneal as a standard control. *C. camphora* oil (CCO) showed significant anti-anxiety and antidepressant effects compared to the control group in rat models. Numerous monoterpenoid compounds in the essential oil of *C. camphora* are confirmed by phytochemical studies. *β*-thujone, *β*-pinene, linalool, and limonene are monoterpenoids that are testified to have antidepressant applications. Furthermore, recent research has proposed the antidepressant action of *β*-pinene, which increases dopamine level and inhibits MAO activity in rabbits. Additionally, few studies showed that numerous biologically active molecules, including monoterpenoids, are potent inhibitors of MAO-A and MAO-B [9] (Figure 8).

#### 9.2.6. Antioxidant Activity

As previously mentioned, *C. camphora* seed kernel oil increased the concentrations of superoxide dismutase and catalase in diet-induced rats, which consequently boosted antioxidant activity and reduced malondialdehyde concentration (a biomarker of lipid peroxidation and oxidative stress) [64].

#### 9.2.7. Analgesic Activity

*C. camphora* leaf essential oils showed a significant analgesic effect against nitroglycerin-induced experimental migraine in mice models and inhibited the nuclear factor-kappa Beta, inducible nitric oxide synthase, and nitric oxide pathway [36].

#### 9.2.8. Hepatoprotective Activity

According to Johari et al. [74], camphor powder solution given female rats by intraperitoneal injection for 14 days showed hepatoprotective activity in the treatment of a deferent type of liver conditions. On the liver enzymes, it is proven to have a stimulating impact. However, the researchers recommended that camphor use in a higher dosage uninterruptedly probably leads to a substantial increase in the concentration of liver enzymes [74].

## 10. Toxicity Studies

*C. camphora essential oil from* seeds, twigs, and leaves showed robust contact toxicity against cotton aphids with median lethal concentration (LC50) values of 146.78, 274.99, and 245.79, mg/L after 48 h of treatment, respectively [37]. Camphor is quickly absorbed by the mucous membranes, skin, and gastrointestinal tract in liquid form. Symptoms may appear 5–90 minutes after consumption [26]. In humans, indications of intoxication include nausea, vomiting, trembling, and convulsions, which are followed by CNS depression characterized by apnea and coma [17, 75].

## 11. Discussion and Comparative Therapeutic Evaluation

Unlike petroleum products, camphor is a botanical hydrocarbon, very inexpensive, and can be easily cultivated without any shortages. Therefore, camphor is an exceptional carbon source for the production of high purity, high yield, and high efficiency [76]. According to Unani physicians, seven factors are responsible for the maintenance of health, and loss of any one of these can lead to disease or even death. Dietotherapy and pharmacotherapy are mainly used to maintain the equilibrium of humors to maintain health and treat disease conditions. All single drugs have specific and many ethnopharmacological properties according to their active principles and temperament. *C. camphora* (L.) is a traditional Unani medicinal plant with a third-degree cold and dry temperament and hence useful in hot temperamental individuals [8] used since ancient times. Nowadays, natural and artificial camphor is also used for medicinal conditions and commercial purposes. A review of Unani and other conventional literature realized that *C. camphora* has prophylactic and several pharmacological properties for treating medical conditions and strengthening mental and physical properties and it is effective in treating respiratory conditions, musculoskeletal, gastrointestinal, oral, eye, integumentary, and general conditions. Unani physicians evaluated ethno-pharmacological properties, usage, patient temperament, and disease condition when prescribing medications and then selected single pharmaceuticals with correctives (*Muslih*) to reduce undesirable or unwanted effects. Furthermore, to combat the adverse effect of camphor on cold temperamental people [12, 15, 16], they advised camphor with hot temperament and fragrance herbs such as *Zafran*, *Amber*, and *Misk* [15].

Camphor's distinctive aroma has led to its widespread use in ointments and inhalants, particularly as a remedy to treat respiratory ailments. Unani physicians stated that *C. camphora* is commonly used in respiratory conditions such as acute and chronic cough, fever, common cold, lung ulcers, pleurisy, pneumonia, coryza, and catarrh in various forms as a single drug or with another herbal, mineral, or animal origin drug as a compound formula as it possesses expectorant, antipyretics, deobstruent, and mucolytic properties [8, 12, 15, 16]. *C. camphora* has been a useful remedy in symptoms of COVID-19 and inhibits SARS CoV-2 spike glycoprotein CoV [53]. Furthermore, one of the ingredients of many compounds, Unani formulation, is useful in viral infections and respiratory diseases (Table 3). In vitro, in vivo, and clinical, recent studies have proven its antibacterial [35, 56, 77], anti-inflammatory [10, 37, 69], analgesics [36], and immunomodulatory properties. Hence, it is beneficial in conditions such as acute non-purulent rhinosinusitis, asthma, COPD, bronchitis, and the inhibitory effect of SARS-CoV [53]. It is used as a nasal decongestant and a cough suppressant [78]. Anti-asthmatic, cough suppressant, mucolytic, bronchodilator control, airway mucus hypersecretion, and anti-asthmatic qualities are found in camphor, cineole, linalool, and safrole [76].

Compound drugs of *C. camphora* and external medication used for the suitable medicament in musculoskeletal conditions such as arthritis and muscular pain with ethnomedicinal properties such as stimulant, rubefacient, anastatic, and analgesics [12]. Camphor is a major active ingredient in liniments and balms used as a topical analgesic, and it is a natural compound [78]. Lee et al. [10] in their in vitro study confirmed anti-inflammatory and antioxidant properties of camphor. They hypothesize that the anti-inflammatory actions of C. camphora is perhaps due to the modulation of cytokine, NO, and PGE(2) production. TRPM8, TRPV3, and TRPV1 (transient receptor potential) channels are activated, and TRPA1 is inhibited by camphor leading to excitation, warm sensation, and desensitization of sensory nerves, itch, relieving pain, and irritation in the applied area [76, 78].

**Figure 9:**
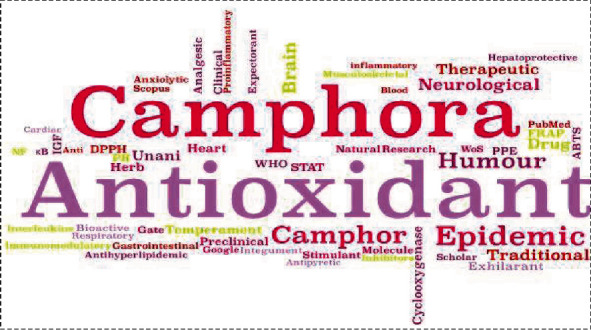
Word cloud of the current study.

**Figure 10:**
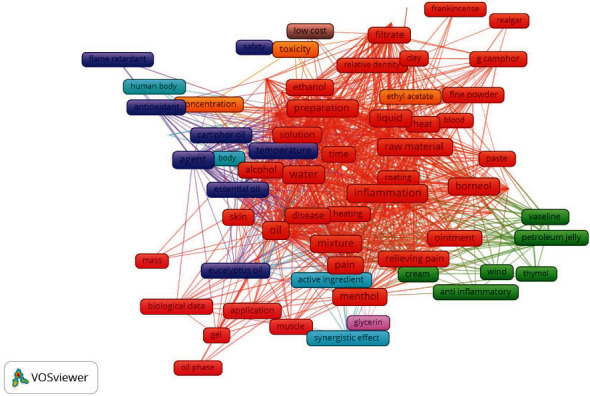
Network visualization of the Camphor based on previously published data in the Web of Science.

We have designed the word cloud in the current study based on the closest terms used in this study (Figure 9). It would be helpful for the researchers, scientists, doctors, students, and academicians to work in this area. Previously, many researchers are used word cloud in the field of stress [79, 80], motor imagery [81], augmented reality [82], blockchain technology [83], and premenstrual syndrome with oxidative stress [84]. In addition, we also designed the network visualization based on previous published studies mentioned in Figure 10. This network visualization may also be helpful to the researchers, scientists, doctors, students, and academicians to work in this area. Previously, many researchers are used network visualization in the field of premenstrual syndrome, insomnia [85], bruxism [86], motor imagery, augmented reality, stress [87], and blockchain technology.

Camphor is a cardiac and nervine tonic in proper dose and dosage forms. It acts as an exhilarant, tonic, stimulant innate faculty of soul and nerves. The Unani formulation such as Jauhar-i-Kafoor is used for inhalation as nervine and exhilarant for vital organs and useful in convulsion. *Jauhar-i-Kafoor* is also useful as cardiac tonic for conditions such as a syncope, weakness of heart functions, and palpitation. Overdose or misused of camphor can cause adverse effects such as abdominal discomfort, tremor, apnea, coma, and CNS depression. Hence, a proper dose stimulates and enhances nervine and cardiac activity, while an overdose can disrupt the system. However, more research is required to confirm. According to the findings, a proper dose stimulates and enhances nervine and cardiac activity, while an overdose can disrupt the system. However, more research is required to confirm. Studies have shown that *C. camphora* or some of its components might find some applications in the future for the treatment of memory disorders or for improving brain functions in patients [76].

## 12. Research Gaps, Future Recommendations, and Concluding Remark

Strength includes authenticity and research articles showed that *C. camphora* has therapeutic applications in various diseases, proven in few recent preclinical and clinical studies. However, other ethnopharmacological activities with eye applications and gastrointestinal and cardiovascular diseases require additional modern pharmacological interpretations to explicate its basic mechanism. The review showed that isolated compounds, extract dose range, route of administration, and dose frequency are clarified in the existing recent research. However, more preclinical and clinical trials are recommended to explore its therapeutic applications in other diseases. Furthermore, it has been normally combined with other single drugs in Unani medicine; hence, drug interactions should be researched further in conventional therapies. There are no studies that have revealed interactions between conventional medicine and camphor, and additional research is needed to determine the safety and efficacy of camphor. Nonetheless, prospective clinical studies are recommended to validate the herb's healing effect. In humans, indications of intoxication include gastrointestinal like nausea, vomiting, and nervous system trembling and convulsions, which are followed by CNS depression characterized by apnea and coma.

The comprehensive review concludes that it is a drug that has been effectively used in Unani medicine for centuries to treat several ailments, particularly respiratory, nervous, musculoskeletal, and eye disorders. Camphor has been related to a variety of biological activities, including antibacterial, antifungal, anti-inflammatory, analgesics, antitussive, and antioxidant. However, bioactivity was often determined using an essential oil rich in camphor rather than pure camphor, and clinical studies of pure camphor have not been found. Moreover, sparse preclinical and clinical researches are available. However, more studies are needed to explore the pharmacological activities. This review has demonstrated that camphor is a medication with many wide ranges of applications.

## Figures and Tables

**Figure 7:**
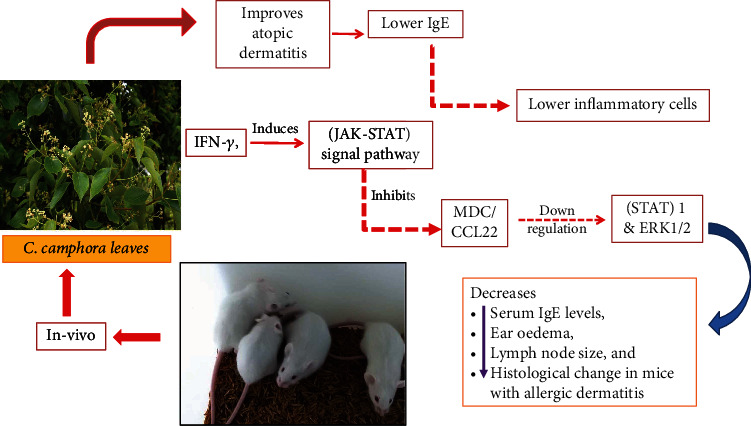
Anti-allergic activity of natural bioactive molecules of *C. camphora* in allergic dermatitis.

**Table 1:** Vernacular name of *C. camphora.*

Language	Vernacular name	Ref.
Unani medicine	*Kafoor*	[14, 16, 23]
Persian	*Kafur, Kafoor*	[14, 16, 23]
English	Camphor, Bheemseni camphor (natural), Bomeo camphor	[14, 16, 23]
Arabic	*Kafoor*	[14, 16, 23]
Germany	Kampher	[14, 16, 23]
French	Camphre	[14, 16, 23]
Hindi	*Duk, Ben, Guj, Kafoof*	[14, 16, 23]
Sanskrit	*Karpoor, Ghausar, Himavaluka*	[14, 16, 23]
Tamil	Pachai Karpooram, Karpooran-Cheena, Karuppuram	[14, 16, 23]
Gujarati	Kapoor, Karpoor	[14, 16, 23]
Telugu	*Pacha Karpooram, Cheen Karpooram*	[14, 16, 23]
Siddha medicine	Karupporam	[13]
Ayurveda medicine	Karpura, Ghanasaara, Chandra, Chandra Praba, Indu, Tushaara, Gandhadravya	[13]

**Table 2:** *C. Camphora* effect on system, therapeutic application, dosage form and method of use.

System	Therapeutic application	Dosage form	Method of usage	Ref.
Respiratory	Pleurisy (*Dhāt al-Janb*) and pneumonia (*Dhāt al-Ri'a*) Epistaxis Lung ulcers Specific fevers (*Hummā Diqq*) Coryza, catarrh (*Nazla-o-Zukām),* and old cough	Oil	External application with suitable oil	[8, 12, 15, 16]

Gastrointestinal	Flatulence (*Riyāh-i-Mi῾da)* Passing loose stools due to the predominance of yellow bile or blood (*Ishāl-i-Safrāwi* and *Damwi)* It can also be used in dysentery, bilious diarrhea, and inflammation of the liver (*Waram al-Kabid)*	—	—	[8, 12, 15, 16]

Musculoskeletal	Joint pain and accumulation of humor in the distended muscle fibers causes soreness (*Waja‘al-Khāsira*, and *Waja‘al-Mafāsil*)	Oil	External application camphor powder mixed with oil	[12]

Integumentary	Skin conditions and reduces irritation	Oil Cream (*Marham*)	External application with suitable oil or cream	[12]

Oral cavity	Tooth pain	Camphor sublimation	Powder or powder with rose oil on affected tooth	[12, 16]
	Stomatitis and toothache (*Qulā‘*and *Dānton ka Dard*)	Gargle and mouth wash	Camphor along with distillate water of *Rosa bourboniana* (*Arq-i-Gulab*)	[15]

Ear condition	Ear pain	Ear drop	Camphor with fresh coriander juice (*Aāb-i-Kasneez Sabs*)	[12, 15]

Eye diseases	Eye irritation Abnormal hot temperament (*Su' Mizaj Hārr)* of eyes. Conjunctivitis and prevents eye involvement in smallpox	Fine powder (*Surma)* Fine powder (*Surma)*	Fine powder apply on eyelid Fine powder with the juice of coriander	[12, 15]

General condition	Fever due to tuberculosis Exhilarant and cardiac tonic	Powder	-	[8, 12, 15, 16]
	Bilious headache, evil heat of soul, and fever	Paste	Location application of paste prepared by mixing powdered camphor with rose oil and grapes apply on the forehead	
	Antidote to scorpion poison	Powder	Powder with rose oil	
	It works in the hot type of inflammation (*Waram-al-Hārrah)* and obstacles (*Sudda-i-Hārr*)	Oil or ointment	Camphor mixed with suitable oil	
	Epistaxis	Nasal drops	Camphor is used with *Myrtus communis* for epistaxis	
	Wound and foot swelling	Dusting powder, ointment	—	

**Table 3:** Unani formulations, dose, dosage form, ethnomedicinal properties, and therapeutic applications of *C. camphora* as one of the ingredients in different body systems.

Unani formulation	Dose	Dosage form	Ethnomedicinal properties	Therapeutic application	Ref.
Respiratory system					
*Habb-i-Nafs-ud-Dam Silli*	5-10 gm	Pills	Hemostyptic (*Habis-i-Dam*), healing agent (*Mudammil)*, Antipyretic (*Dafi‘-i-Humma*)	Nasal bleeding (*Nafs-ud-Dam*), phthisis (*Sill*), asthma (*Diq al-Rewi*)	[31]
*Habb-i-Jawahar Muwallif Khas*	500 mg	Pills	Tonic (*Muqawwi*), expectorant (*Munaffis-i-Balgham*), analgesics (*Musakkin*)	Phthisis, bronchial asthma	[32]
*Habb-i-Khunaq*	5-10 gm	Pill	Analgesics, anti-inflammatory (*Muhallil-i-warm*)	Inflammation of bilateral pharyngeal muscle/diphtheria (*Khunaq*), pharyngitis, and laryngitis	[32]
Gastrointestinal system					
*Habb-i-Qabid*	125-250 mg	Pills	Astringent (*Qabid*), antiseptic (*Dafi‘-i-Ta‘affun*)	Infantile diarrhoea (*Ishal al-Atfal*)	[33]
*Qurs-i-Atash*	5-10 gm	Tablet	Anti-bilious *(Dafi‘-i-Safra)*	Reduce thirst, acidosis *(Humudat-i-Mi'da)*	[31]
*Qurs-i-Kafoor Musik*	5-10 gm	Tablet	Astringent	Diarrhea	[31]
*Jawarish-i-Kafoor*	5-10 gm	Semisolid confection	Stomachic (*Muqawwi-i-Mi'da*), carminative (Kāsir-i-Riyāh)	Dyspepsia (*Su' al-Hadm*) indigestion (*Tukhma*), flatulence (*Nafkh-i-Shikam*)	[31]
*Habb-i-Taoon Qawi*	250-500 gm	Pill	Antidote *(Dafi‘-i-sumum*)	Plague (*Ta‘un*) Food poisoning (*Hayda*)	[32]
*Habb-i-Taiyub-ul-fam*	5-10 gm	Pill	*Mutib-i-Dehan*	Halitosis (*Bakhr al-Fam*)	[32]
*Safuf-i-kahruba*	3-5gm	Powder	Digestive (*Hazim*), laxative (*Mulaiyin*), appetizer (*Mustahi*)	Indigestion *(Du'f‘-i-hadim*), reduce thirst (*Du‘f-i-*istiha)	[23]
*Habb-e- Pechish*	220 mg (one pill)	Pill	Astringent and hemostyptic	Dysentery, diarrhea	[23]
*Qurs-e-Zaheer*	2 tab twice	Tablet	Anti-dysenteric, stomachic	Dysentery, indigestion (*Dafi‘-i-Mi'da)*	[34]
*Tiryaq-i-Pechish Jadid*	3 gm twice a day	Powder	Anti-diarrhea	Useful in bilious and phlegmatic dysentery (*Zahīr Safrawi*), *Wa Balghami)*, Chronic dysentery (*Zahīr Muzmin*)	[34]
*Marham-i-Bawaseer*	Q.S. for external use	Ointment	Prevent piles *(Dafi‘-Bawasīr)*	Burning sensation of hemorrhoid (*Sozish-i-Bawasīr*), painful hemorrhoid (*Waj-ul-Bawasīr*), bleeding piles (*Bawasīr Damya)*	[34]
*Marham Saeeda Chob Neemwal*	Q.S for external us	Ointment	Anti-inflammatory	Hemorrhoid	[31]
Eye					
*Jauhar-i-Naushadar*	Q.S	Semisolid sublimation	Detergent (*Jālī*), analgesics	Keratitis (*Sabal*), corneal opacity (*Bayād*), pterygium (*Zufra*) (for ophthalmic use)	[31]
*Kohal-i-Kafoor*	Q.S	Finest powder (for ophthalmic use)	Resolvent (*Muhallil-i-Waram*)	Conjunctivitis, burning sensation of eye	[31]
*Burood-i-Muqawwi-i-Basar*	Q.S	Fine powder (for ophthalmic use)	Eye tonic (*Muqawwi-i-Basar*)	Asthenopia/amblyopia (*Dafi‘*-al-*Basar*)	[31]
*Burood-i-Sozish-i-Chashm*	Q.S	Fine powder (for ophthalmic use)	Refrigerant (*Mubarrid*), analgesics	Burning sensation of the eye (*Sozish-i-Chashm),* eye irritation (*Kharish-i-Chashm*)	[31]
*Kohlul Jawahar*	Q.S	Past	Eye tonic	Weakness eyesight *(Duf‘-i-Basar*)	[31]
Cardiovascular system					
*Mufarrah Shaikh ur Rais*	5 gm	Semisolid confection	Cardiac tonic (*Muqawwi-i-Qalb*)	Weakness of heart *(Du'f al Qalb*) and palpitation (*Khafqan*)	[31]
Integumentary system					
*Marham Kharish Jadeed*	Q.S for external us	Ointment	Refrigerant and antibacterial (*Qatil-i-Jara'sim*)	Fungal infection (*Dād*), ringworm (*Qūbā*), irritation (*Kharish*), itching (*Hikkah*), and blood infection (*Fasad-i-dam*)	[31]
*Marham Kafoor*	Q.S for external us	Ointment	Refrigerant, antiseptic	Ulcer (*Qurūh)* and inflammatory wound	[31]
*Marham-e-Safaida Kafoori*		Ointment	Healing agent (*Mudammil*), antiseptic (*Dafi‘-i-Ta‘affun*), wound (*Qurooh-i-Afni*)	Wound	[31]
Reproductive system					
*Jauhar-i-Kafoor*	125 mg in a capsule	Dried powder	Antiseptic, refrigerant (*Mubarrid*)	Gonorrhoea (*Sozāk*)	[31]
*Halwa-i-Suparipak*	10-20 gm	Semisolid preparation	Spermatogenic (*Muwalliz-i-Mani*), retentive of semen (*Mumsik*), aphrodisiac (*Muqawwi-i-Bah*)	Spermatorrhoea (*Jarayan*), nocturnal emersion (*Surat-i-Inzal*), loss of libido (*Du'f al Bah*)	[31]
Nervous system					
*Jauhar-i-kafoor kawi*	For inhalation	Semisolid sublimation	Nervine tonic (*Muqawwi dimagh*)	Convulsion (*Sara*), syncope (*Ghazhi*)	[32]
Miscellaneous					
*Qurs-i-Sartan-Kafoori*	3-5 gm	Tablet	Hemostyptic, antipyretic	Type of bilious fever with excessive thirst and bilious vomiting *(Hummā -i-Muharraqa*), tuberculosis (*Dīq*), cough, phthisis	[31]
*Qurs i-kafoor Lulvi*	2-4 gm	Tablet	Antipyretic, healing agent, expectorant, astringent	Acute fever (*Hummā al-hadda*), phthisis, hectic fever (*Hummā diq*), gastrogenic diarrhea (*Ishal mi‘di*)	[32]
*Habb-i-kafoor marwareed*	500 mg	Pills	Antidote, antipyretic	Fever, epidemic fever (*Hummā- Waba'iyya*)	[32]
*Hab-i-Tap-i-larza*	150-250 mg	Pill	Antipyretic	Type of fever *(Hummā -i-Ejamia*)	[32]
*Hab-i-kafoor*	125-250 mg	Pill	Antipyretic	*Hummā -i-muharraqa*	[23]
*Qur-i-kafoor*	Four tablets (each 775 mg)	Tablet	Refrigerant	Hectic fever and bilious fever	[31]
*Habb-i-Ikseer Bukhar*	400 mg thrice a day with lukewarm water	Pill	Antipyretic for fever with chills (*Dafi‘-tap-i-Larza*) Antipyretic for seasonal fever *(Dafi‘-i-tap-i-Mausami)*	Continuous fever Seasonal fever	[34]

Q.S.: quantity as required.

**Table 4:** Natural bioactive molecules found in different parts of the *C. camphora* tree.

Part of the tree	Major natural bioactive molecules												
	Camphor	1,8-Cineole	Linalool	Citronellal	*α*-pinene	Camphene	Safrole	*β*-pinene	Limonene	Eucalyptol	*α*-Terpineol or terpineol	D-borneol	References
Leaf oil	+	+	+	+	+	+							[18]
Branch	+				+	+							[18]
Wood essential oil	+	+					+				+		[18]
Root essential oil	+						+				+		[18]
Essential oil from leaf and branch mixture	+	+			+	+		+	+		+		[18, 38]
Essential oil from wood, leaf and branch mixture	+				+				+		+		[18]
Twig essential oil	+									+			[37]
Seeds oil	+		+							+			[37]
Fruit oil	+		+				+						[37, 39]
Fresh leaves												+	[10, 40]

**Table 5:** Preclinical (*in vitro/in vivo/*in silico) and clinical studies of *C. camphora* and its main natural bioactive molecules.

Natural bioactive molecule	Methodology	Test drug	Control	Result	Pharmacological activity /disease	Ref.
Camphor	Cell line study (male Wister rat DRG cells)	Dorsal root ganglion of adult male Wistar rats	Crt wash	Activate TRP receptor (TRPM8) and mutant channel	Analgesic by cold and heat sensitization of camphor	[50]
Citronellal	Rat module *in vivo* and *in vitro* (paw edema and peritoneal fluid leucocyte count)	50,100,200 mg/kg	Dexamethasone (2 mg/kg, s.c.),	In vitro and vivo studies of citronellal significantly (*p* < 0.01) reduce paw edema and leucocyte count	Anti-inflammatory and analgesics	[47]
*Clinical trials*						
Cinelol	Randomized, double-blind, clinical trial	100 mg capsule three daily for 7 days	Placebo	Symptom (headache, heaviness, secretion, and nasal obstruction) reduced	Acute non-purulent rhinosinusitis	[42]
Cineole	Randomized placebo-controlled trial (multicenter)	200 mg cineole or placebo 3 times for six months during (concomitant therapy)	Placebo	Significant improvement notes test group Improvement in dyspnea, lung function	Anti-asthmatic	[43]
Cineole (Eucalyptol)	Randomized placebo controlled clinical trial	200 mg cineole or placebo 3 times for six months during winter	Placebo	Improve lung function and health status. Reduce exacerbation and dyspnea	COPD	[44]
Cinelol	Randomized placebo-controlled clinical trial	3 × 200 mg of cineole, per day for 10 days	Placebo control	Significantly reduce cough *p* = 0.0001	Bronchitis	[41]

**Table 6:** Unani compound formulations with *C. camphora* and pharmacological application in preclinical (*in vitro/in vivo/*in silico) and clinical studies.

Unani formulation	Method/model	Extract use/dosage form	Control/organism tested	Result	Pharmacological application	Ref.
*Preclinical studies*						
Arq-Ajeeb	*In vivo* (rats)	0.07 ml and 0.14 ml/kg, p.o.	Charcoal administration	Reduce diarrhea in rats	Anti-diarrheal activity	[51]
Compound preparation of sesame oil, camphor, and honey	Animal (rat)	Daily dressing with extract	Oil Vaseline	Maximal healing was noticed in the test group	Healing effect of second-degree burn	[52]
Arq Ajīb contains methanol, camphor	In silico approach	Inhibits SARS-CoV-2 spike glycoprotein and main protease	—	Good interactions and binding affinities with 3CLpro and S glycoprotein	Inhibitory effect on SARS-CoV-2	[53]
Extract C. camphora and Ziziphora tenuior	In vitro mice liver	3, 5, 10, 25, 50, and 100 mg/ml of extracts	—	The extract exhibited dose-dependent and time-dependent antiparasitic effects	Anti-parasitic and immunomodulatory	[54]
*Clinical studies*						
Marham-i-Raal	Single-arm pre- and posttreatment study	2 gm on episiotomy wound	—	REEDA score decreased, and VAS score decreased	Episiotomy wound healing and pain reduction	[55]
Marham-i-Raal	Case study	External application (3 months) (ointment)	—	Completely heal foot ulcer	Chronic wound healing	[21]
Arq Ajib	Clinical study	Liquid application	—	Decrease in VAS score for pain intensity	Headache	[56]
Composition of A. indica and C. camphora	Controlled clinical trial	Oral and topical	Psoralen plus ultraviolet A (PUVA) solution	In individuals with moderate-to-severe CPP, test medications that are efficacious and well-tolerated	Chronic plaque psoriasis	[57]

## Data Availability

The data will be available with the first author.

## Abbreviations

ABTS: 2,2′-Azino-Bis 3-ethylbenzothiazoline-6-sulfonic acid
BC: Before Christ
COPD,: Chronic obstructive pulmonary disease
COX: Cyclooxygenase
DPPH: 2,2-diphenyl-1-picryl-hydrazyl hydrate
DM: Diabetes mellitus
CNS: Central nervous system
CCEO: *C. camphora* essential oil
FRAP: Ferric reducing antioxidant power
IGF-I: Insulin-like growth factor-I
IL-1: Interleukin-I
IL-6: Interleukin-6
iNOS: Inducible nitric oxide synthase
LPG/IFN: Interferons
MAO-A: Monoamine oxidase a
MeOH: Methanol
MBC: Methyl benzylidene camphor
mRNA: Messenger RNA
MMP1: Matrix metalloproteinase-1
MSP: *M. oleifera* seed preparation
NIUM: National Institute of Unani medicine
NO: Nitric oxide
NOS,: Nitric oxide synthase
NF-Kb: Nuclear factor-kappa B
PGE2: Prostaglandin E2
PPE: Preproenkephalin
PR: Progesterone receptor
PUVA: Psoralen plus ultraviolet a
REEDA: Redness, edema, ecchymosis, discharge, approximation
TRPV1: Transient receptor potential A1
TRPM8: Transient receptor potential cation channel subfamily M (melastatin), member 8
TNF-*α*: Tumor necrotic factor alpha
UV: Ultra violet
VAS: Visual analogue scale
JAK-STAT: Janus kinase signal transducer and activator of transcription
MDC/CCL22: Macrophage-derived chemokine
GMP: Guanosine triphosphate
GTP: Guanosine-5′-triphosphate.

## References

[1] WHO (2019). *WHO Global report on traditional and complementary medicine 2019*.

[2] Mehdi S., Sultana A., Heyat M. B. B. (2022). A review of amenorrhea toward Unani to modern system with emerging technology: current advancements, research gap, and future direction. *Computational Intelligence in Healthcare Applications*.

[3] Sultana A., Mehdi S., Rahman K. (2022). Recent advancements of pelvic inflammatory disease: A review on evidence-based medicine. *Computational Intelligence in Healthcare Applications*.

[4] Ansari S., Khan Q. A., Anjum R., Siddiqui A., Sultana K. (2017). Fundamentals of Unani system of medicine - a review. *European Journal of Biomedical and Pharmaceutical Science*.

[5] Ahmed S. I. (1980). *Introduction to Al-Umoor Al-Tabiyah*.

[6] Kitab R. M. Z. (2001). *Ul-Mansoori (Urdu Translation)*.

[7] Sultana A., Baig, Rahman (2022). Contemporary overview of bacterial vaginosis in conventional and complementary and alternative medicine. *Computational Intelligence in Healthcare Applications*.

[8] Baghdadi H. (2005). *Kitab ul-Mukhtarat Fi’l Tibb*.

[9] Jay Rabadia N. V., Satish S., Ramanjaneyulu J. (2013). An investigation of anti-depressant activity of Cinnamomum Camphora oil in experimental mice. *Asian Journal of Biomedical and Pharmaceutical Sciences*.

[10] Lee H. J., Hyun E. A., Yoon W. J. (2006). In vitro anti-inflammatory and anti-oxidative effects of Cinnamomum camphora extracts. *Journal of Ethnopharmacology*.

[11] Singh R., Jawaid T. (2012). Cinnamomum camphora (Kapur): review. *Pharmacognosy Journal*.

[12] Kabir al-Din M. (2007). *Makhzan al-Mufradat*.

[13] Khare C. P. (2007). *Indian Medicinal Plants: An Illustrated Dictionary (Google eBook)*.

[14] Nadkarni K. M. (2010). *Indian Plants and Drugs*.

[15] Khan M. (2018). *Muhit-i-A’zam*.

[16] Abdul Hakim M. H. (2002). *Bustan ul-Mufridat*.

[17] Hausner E. A., Poppenga R. H. (2013). *Hazards Associated with the Use of Herbal and Other Natural Products*.

[18] Kirtikar B., Basu (2012). *Indian Medicinal Plants*.

[19] Garg N., Jain A. (2015). Therapeutic and medicinal uses of Karpura-a review. *International Journal of Science and Research*.

[20] Tewari G. (2017). A review on aroma profile of Cinnamomum species in North and North East India. *World Journal of Pharmaceutical Reseaech*.

[21] Alam S. S., Ahmad W., Rizwanullah M., Muzammil M. (2020). Healing of a traumatic wound with herbo-medicinal ointment; Marham-E-Raal: a case study. *Journal of Pharmaceutical and Scientific Innovation*.

[22] Zuccarini P., Soldani G. (2009). Camphor: benefits and risks of a widely used natural product. *Acta Biologica Szegediensis*.

[23] Anonymus (2011). *National Formulary of Unani Medicine. th Part 6 ., Part IV*.

[24] Sultana A., Begum W., Saeedi R. (2022). Experimental and computational approaches for the classification and correlation of temperament (Mizaj) and uterine dystemperament (Su’-I-Mizaj Al-Rahim) in abnormal vaginal discharge (Sayalan Al-Rahim) based on clinical analysis using support vector Mach. *Complexity*.

[25] Narayan S., Singh N. (2012). Camphor poisoning-an unusual cause of seizure. *Medical Journal, Armed Forces India*.

[26] Barrueto F. (2005). *Camphor*.

[27] Ansari A. P., Sana S. H., Ansari H. (2020). The concept of Abd ā l-i-Adwiya (drug substitution / therapeutic interchange) in Unani medicine: a critical appraisal. *Journal of Advanced Research in Pharmaceutical Sciences and Pharmacology Interventions*.

[28] Razi A. (1999). *Kitab al-Abdāl*.

[29] Sina I. (2010). *Al-Qanun fi’l Tibb*.

[30] Ghani N. (2001). *Khazainul Advia Vol. I-IV*.

[31] Anonymus (2011). *National Formulary of Unani Medicine. th Part 6., Part II*.

[32] Anonymus (2011). *National Formulary of Unani Medicine. th Part 6 ., Part III.V*.

[33] Anonymus (2011). *National Formulary of Unani Medicine. th Part 6 ., Part VI*.

[34] Anonymus (2011). *National Formulary of Unani Medicine. th Part 6 ., Part I*.

[35] Poudel D. K., Rokaya A., Ojha P. K. (2021). The chemical profiling of essential oils from different tissues of *Cinnamomum camphora* L. and their antimicrobial activities. *Molecules*.

[36] Fan L. Y., Lin Q., Yang N. Y., Chen L. H. (2020). Analgesic effects of the essential oil from *Cinnamomum camphora* against nitroglycerin-induced migraine in mice. *Indian Journal of Pharmaceutical Sciences*.

[37] Jiang H., Wang J., Song L. (2016). Gc×Gc-tofms analysis of essential oils composition from leaves, twigs and seeds of *Cinnamomum camphora* l. presl and their insecticidal and repellent activities. *Molecules*.

[38] Joshi S. C., Padalia R. C., Bisht D. S., Mathela C. S. (2009). Terpenoid diversity in the leaf essential oils of Himalayan Lauraceae species. *Chemistry & Biodiversity*.

[39] Jianyu S. (2012). Composition and biological activities of the essential oil extracted from a novel plant of *Cinnamomum camphora* Chvar. Borneol. *Journal of Medicinal Plant Research*.

[40] Lee S. H., Kim D. S., Park S. H., Park H. (2022). Phytochemistry and applications of *Cinnamomum camphora* essential oils. *Molecules*.

[41] Fischer J., Dethlefsen U. (2013). Efficacy of cineole in patients suffering from acute bronchitis: a placebo-controlled double-blind trial. *Cough*.

[42] Kehrl W., Sonnemann U., Dethlefsen U. (2004). Therapy for acute nonpurulent rhinosinusitis with cineole: results of a double-blind, randomized, placebo-controlled trial. *The Laryngoscope*.

[43] Worth H., Dethlefsen U. (2012). Patients with asthma benefit from concomitant therapy with cineole: a placebo-controlled, double-blind trial. *The Journal of Asthma*.

[44] Worth H., Schacher C., Dethlefsen U. (2009). Concomitant therapy with cineole (Eucalyptole) reduces exacerbations in COPD: a placebo-controlled double-blind trial. *Respiratory Research*.

[45] Wang S., Zhang D., Hu J. (2017). A clinical and mechanistic study of topical borneol-induced analgesia. *EMBO Molecular Medicine*.

[46] Karashima Y., Damann N., Prenen J. (2007). Bimodal action of menthol on the transient receptor potential channel TRPA1. *The Journal of Neuroscience*.

[47] Melo M. S., Guimarães A. G., Santana M. F. (2011). Anti-inflammatory and redox-protective activities of citronellal. *Biological Research*.

[48] Maerkel K., Durrer S., Henseler M., Schlumpf M., Lichtensteiger W. (2007). Sexually dimorphic gene regulation in brain as a target for endocrine disrupters: developmental exposure of rats to 4-methylbenzylidene camphor. *Toxicology and Applied Pharmacology*.

[49] Yang H., Xu P., Song W., Zhai X. (2021). Anti-tyrosinase and antioxidant activity of proanthocyanidins from *Cinnamomum camphora*. *International Journal of Food Properties*.

[50] Selescu T., Ciobanu A. C., Dobre C., Reid G., Babes A. (2013). Camphor activates and sensitizes transient receptor potential melastatin 8 (TRPM8) to cooling and icilin. *Chemical Senses*.

[51] Khan M. A., Khan N. A., Qasmi I. A., Ahmad G., Zafar S. (2004). Antidiarrhoeal activity of Arque-Ajeeb, a compound formulation of Unani medicine in rats. *Oriental Pharmacy and Experimental Medicine*.

[52] Vaghardoost R., Majd S. G., Tebyanian H. (2018). The healing effect of sesame oil, camphor and honey on second degree burn wounds in rat. *World Journal of Plastic Surgery*.

[53] Ahmed N. Z., John Davis G. D., Khan A. A. (2022). Arq Ajīb - a wonder Unani formulation for inhibiting SARS-CoV-2 spike glycoprotein and main protease - an *in silico* approach. *Journal of Complementary and Integrative Medicine*.

[54] Kanaan M. H. G., Anah S. A., Jasim G. A., Ghasemian A. (2021). In-vitro protoscolicidal and immunomodulatory effects of *Cinnamomum camphora* and Ziziphora tenuior against Echinococcus granulosus protoscolices. *Reviews in Medical Microbiology*.

[55] Sultana A., Joonus A. F., Rahman K. (2021). Effect of Marham-i-Raal on episiotomy wound healing: a single-arm pre-and post-treatment study. *Cellmed Orthocellular Medicine and Pharmaceutical Association*.

[56] Zafar M. M. I., Hassan F., Naqvi S. B. S. (2019). Evaluation of antibacterial activity of camphor, benzoin, cubebs, fenugreek, apricot and cinnamon leaf against standard cultures and clinical isolates of an array of organisms, Pakistan. *Journal de Pharmacologie*.

[57] Khanna N., Nazli T., Siddiqui K. M., Kalaivani M., Rais-ur-Rahman (2018). A non-inferiority randomized controlled clinical trial comparing unani formulation & psoralen plus ultraviolet a sol in chronic plaque psoriasis. *The Indian Journal of Medical Research*.

[58] Mahdi I., Bakrim W. B., Bitchagno G. T. M., Annaz H., Mahmoud M. F., Sobeh M. (2022). Unraveling the phytochemistry, traditional uses, and biological and pharmacological activities of Thymus algeriensis Boiss. & Reut. *Oxidative Medicine and Cellular Longevity*.

[59] Guo Q., Li F., Duan Y. (2020). Oxidative stress, nutritional antioxidants and beyond. *Science China. Life Sciences*.

[60] Hussain T., Murtaza G., Metwally E. (2021). The role of oxidative stress and antioxidant balance in pregnancy. *Mediators of Inflammation*.

[61] Hussain T., Tan B., Murtaza G. (2020). Flavonoids and type 2 diabetes: Evidence of efficacy in clinical and animal studies and delivery strategies to enhance their therapeutic efficacy. *Pharmacological Research*.

[62] Muhamad S. H. A., On S., Sanusi S. N. A., Hashim A. A., Addinna Zai M. H. (2019). Antioxidant activity of camphor leaves extract based on variation solvent. *Journal of Physics Conference Series*.

[63] Liu Z., Kong L., Lu S., Zou Z. (2019). Application of a combined homogenate and ultrasonic cavitation system for the efficient extraction of flavonoids from *Cinnamomum camphora* leaves and evaluation of their antioxidant activity in vitro. *Journal of Analytical Methods in Chemistry*.

[64] Fu J., Zeng C., Zeng Z., Wang B., Gong D. (2016). *Cinnamomum camphora* seed kernel oil ameliorates oxidative stress and inflammation in diet-induced obese rats. *Journal of Food Science*.

[65] Wang L., Zhang K., Zhang K. (2020). Antibacterial activity of Cinnamomum camphora essential oil on Escherichia coli during planktonic growth and biofilm formation. *Frontiers in Microbiology*.

[66] Kumar K. A., Choudhary R. K., Joshi B., Ramya V., Sahithi V., Veena P. (2011). Determination of antibacterial, antifungal activity and chemical composition of essential oil portion of unani formulation kulzam. *International Journal of Green Pharmacy*.

[67] Ho C., Wang E. I. (2009). Essential Oil Compositions and Bioactivities of the Various Parts of *Cinnamomum camphora* Sieb. var. linaloolifera Fujuta. *Forestry Research Quarterly*.

[68] Mishra A. K., Dwivedi S. K., Kishore N., Dubey N. K. (1991). Fungistatic properties of essential oil of Cinnamomum camphora. *Pharmaceutical Biology*.

[69] Kang N. J., Han S. C., Yoon S. H. (2019). Cinnamomum camphora leaves alleviate allergic skin inflammatory responses in vitro and in vivo. *Toxicology Research*.

[70] Azhunova T. A., Nikolaev S. M., Buraeva L. B., Dashiev D. B., Banzaraksheeva S. A. (2009). Hypolipidemic, anti-oxidant and anticoagulant action of camphor-25 compound. *Patologicheskaia Fiziologiia i Èksperimental'naia Terapiia*.

[71] Jadhav M. V., Sharma R. C., Mansee R., Gangawane A. K. (2010). Effect of Cinnamomum camphora on human sperm motility and sperm viability. *Journal of Clinical Research Letters*.

[72] Sen P. K., Garg S. (2019). Wound repair and regenerating effect of ethyl acetate soluble fraction of ethanolic extract of Cinnamomum camphora leaves in wistar albino rats. *Journal of Drug Delivery and Therapeutics*.

[73] Dawood T. (2016). Effect of adding Cinnamomum camphora on the testosterone hormone and reproductive traits of the Awassi rams. *Kufa Journal For Veterinary Medical Sciences*.

[74] Johari H., Abedini M., Fallahi S. (2015). The effect of camphor (Cinnamomum camphora) on concentration of liver enzymes in female rats. *International Journal of Latest Research in Science and Technology*.

[75] Poppenga R. H. (2006). *Hazards Associated with the Use of Herbal and Other Natural Products*.

[76] Hamidpour R., Hamidpour S., Hamidpour M., Hamidpour R. (2019). The Effect of Camphor Discovery for Treating Asthma. *Advances in Bioengineering and Biomedical Science Research*.

[77] Li Y., Zhang Q. Y., Sun B. F. (2021). Single-cell transcriptome profiling of the vaginal wall in women with severe anterior vaginal prolapse. *Nature Communications*.

[78] Xu H., Blair N. T., Clapham D. E. (2005). Camphor activates and strongly desensitizes the transient receptor potential vanilloid subtype 1 channel in a vanilloid-independent mechanism. *The Journal of Neuroscience*.

[79] Akhtar F., Patel P. K., Heyat M. B. B. (2022). Smartphone addiction among students and its harmful effects on mental health, oxidative stress, and neurodegeneration towards future modulation of anti-addiction therapies: a comprehensive survey based on SLR, research questions, and network visualization. *CNS & Neurological Disorders - Drug Targets*.

[80] Teelhawod B. N., Akhtar F., Heyat M. B. B. Machine learning in E-health: a comprehensive survey of anxiety.

[81] Guragai B., AlShorman O., Masadeh M., Heyat M. B. B. A Survey on Deep Learning Classification Algorithms for Motor Imagery.

[82] Sheikh S., Heyat M. B., AlShorman O., Masadeh M., Alkahatni F. A review of usability evaluation techniques for augmented reality systems in education.

[83] Akhtar F., Li J. P., Heyat M. B. Potential of blockchain technology in digital currency: a review.

[84] Sultana A., Rahman K., Heyat M. B., Akhtar F., Muaad A. Y. (2022). Role of inflammation, oxidative stress, and mitochondrial changes in premenstrual psychosomatic behavioral symptoms with anti-inflammatory, antioxidant herbs, and nutritional supplements. *Oxidative Medicine and Cellular Longevity*.

[85] Bin Heyat M. B., Akhtar F., Ansari M. A. (2021). Progress in detection of insomnia sleep disorder: a comprehensive review. *Current Drug Targets*.

[86] Heyat M. B., Akhtar F., Khan M. H. (2021). Detection, treatment planning, and genetic predisposition of bruxism: a systematic mapping process and network visualization technique. *CNS & Neurological Disorders Drug Targets*.

[87] Akhtar F., Heyat M. B., Li J. P., Patel P. K., Guragai B. Role of machine learning in human stress: a review.

